# p53 β-hydroxybutyrylation attenuates p53 activity

**DOI:** 10.1038/s41419-019-1463-y

**Published:** 2019-03-11

**Authors:** Kun Liu, Fangzhou Li, Qianqian Sun, Ning Lin, Haichao Han, Kaiqiang You, Feng Tian, Zebin Mao, Tingting Li, Tanjun Tong, Meiyu Geng, Yingming Zhao, Wei Gu, Wenhui Zhao

**Affiliations:** 1 Department of Biochemistry and Molecular Biology, Beijing Key Laboratory of Protein Posttranslational Modifications and Cell Function, Peking University Health Science Center, 38 Xueyuan Road, 100191 Beijing, China; 2 Department of Biomedical Informatics, Beijing Key Laboratory of Protein Posttranslational Modifications and Cell Function, Peking University Health Science Center, 38 Xueyuan Road, 100191 Beijing, China; 3 Department of Laboratory Animal Science, School of Basic Medical Sciences, Beijing Key Laboratory of Protein Posttranslational Modifications and Cell Function, Peking University Health Science Center, 38 Xueyuan Road, 100191 Beijing, China; 4 Department of Pharmacology I, Shanghai Institute of Materia Medica, 555 Zu Chong Zhi Road, Zhang Jiang Hi-Tech Park, 201203 Pudong, Shanghai China; 5 Ben May Department of Cancer Research, The University of Chicago, Chicago, IL USA; 6 Institute for Cancer Genetics, and Department of Pathology and Cell Biology, College of Physicians and Surgeons, Columbia University, 1130 St. Nicholas Avenue, New York, NY 10032 USA

## Abstract

p53 is an essential tumor suppressor, whose activity is finely tuned by the posttranslational modifications. Previous research has reported that β-hydroxybutyrate (BHB) induces β-hydroxybutyrylation (Kbhb), which is a novel histone posttranslational modification. Here we report that p53 is modified by kbhb and that this modification occurs at lysines 120, 319, and 370 of p53. We demonstrate that the level of p53 kbhb is dramatically increased in cultured cells treated with BHB and in thymus tissues of fasted mice, and that CBP catalyze p53 kbhb. We show that p53 kbhb results in lower levels of p53 acetylation and reduced expression of the p53 downstream genes p21 and PUMA, as well as reduced cell growth arrest and apoptosis in cultured cells under p53-activating conditions. Similar results were observed in mouse thymus tissue under starvation conditions, which result in increased concentrations of serum BHB, and in response to genotoxic stress caused by γ-irradiation to activate p53. Our findings thus show that BHB-mediated p53 kbhb is a novel mechanism of p53 activity regulation, which may explain the link between ketone bodies and tumor, and which may provide promising therapeutic target for cancer treatment.

## Introduction

The p53 protein is one of the most widely studied transcription factors. The *TP53* gene (in mice is *Trp53* gene) has long been recognized as a vitally important tumor suppressor gene because it is mutated and inactivated in more than 80% of human cancer cases^1^. p53 acts as the core node of a complicated and finely tuned network by which it controls and regulates cellular responses to various endogenous and extraneous stressors, and maintains intracellular homeostasis^2–5^. When a stress signal is transduced to p53, its activity is finely tuned by mechanisms that include modulation of protein stability, coactivator and inhibitor recruitment, and posttranslational modifications such as acetylation, methylation, phosphorylation, ubiquitination, sumoylation, and neddylation^2–4^. Activated p53 induces the transcription of various target genes and microRNAs involved in cellular processes such as cell growth arrest, apoptosis, autophagy, ferroptosis, senescence, aging, and metabolism, including the maintenance of oxidative balance^2–8^.

The major ketone bodies are β-hydroxybutyrate (BHB) and acetoacetate, which can be converted into each other and trace amounts of acetone. Ketone bodies are predominantly formed in the liver by acetyl coenzyme A, which degrades fatty acids via β-oxidation. Ketone bodies are the normal fuel for respiration and act as important sources of energy for the heart and brain during starvation^9–11^. In addition to serving as an energy source, ketone bodies are also increasingly recognized as factors that fulfill signaling roles in cellular homeostasis^12–17^ (reviewed in refs. ^18–20^). Ketone bodies are also linked to cancer. For example, they reduced pancreatic cancer growth in mouse xenograft models^21^, and decreased the proliferation and viability of the highly metastatic VM-M3 cells, and prolonged the survival of VM-M3 xenograft mice^22^. The ketone body, acetoacetate, also selectively induces HMGCL expression, enhances the interaction between BRAF V600E and MEK1, and amplifies MEK-ERK signaling to drive tumor cell proliferation and growth in melanoma^23^. The use of ketogenic diets and calorie restriction also have therapeutic effects in human and mouse brain tumors^24^.

β-hydroxybutyrylation (kbhb) is a novel histone BHB-mediated posttranslational modification. Histone kbhb has been detected in yeast, flies, mice, and human cells^13^, and a total of 44 histone kbhb sites have been identified in both human cells and in mouse livers^13^. H3K9 kbhb is enriched in active gene promoters and is associated with genes upregulated in the starvation-responsive pathway^13^. These genes are distinct to those marked by H3K9ac or H3K14me3^13^. In human cells, histone kbhb levels increase following treatment with BHB^13^. Histone kbhb is also significantly induced in the mouse liver by starvation or by streptozotocin-induced diabetic ketoacidosis under conditions of increased plasma BHB levels^13^.

Till now, kbhb has been described only in histone proteins, but none of them in nonhistone proteins, particularly in transcription factors. It is well-known that almost every kind of posttranslational modification that takes place in histones also occurs in p53 protein. Our prior evidence from mass spectrometry analysis data suggested that p53 may be β-hydroxybutyrylated. Although several posttranslational regulatory mechanisms have been described in p53, the role of kbhb in the regulation of this important tumor suppressor protein has not yet been investigated. Therefore, here we studied p53 kbhb. p53 kbhb is an entirely novel discovery. We report that p53 undergoes kbhb at three main lysine residues: lysines 319, 120, and 370, as identified by mass spectrometry and confirmed by site mutation. Our findings show that CBP/p300 catalyzes p53 kbhb, in vitro and in vivo, and that CBP mutants, which occur naturally in lymphoma, exhibit decreased p53 kbhb activity. p53 kbhb attenuates p53 acetylation levels, as well as the transcriptional activity of p53 at canonical p53 target genes, including p21 and PUMA, thereby reducing the effects of p53 on cell apoptosis and cell growth. We propose from our findings that p53 kbhb is a novel mechanism by which ketone bodies have oncogenic roles.

## Methods

### Antibodies and plasmids

The following antibodies were used in western blot assays: anti-β-actin (A15), anti-Flag M2 and anti-Flag M2 agarose resin (Sigma), anti-HA (3F10), anti-HA agarose resin (Roche Applied Science), anti-p53 (DO-1, which detects the N-terminal epitope comprising amino acids 11–25), anti-p53 (full-length), anti-CBP(Cell Signaling Technology), anti-p21, anti-PUMA, anti-MDM2 (Santa Cruz), anti-Ac-K101-p53, anti-Ac-K164-p53, anti-Ac-p53 C-terminal (made in-house), anti-pan-acetylation-Lysine (Ac-K) and anti-pan β-hydroxybutyrylation-Lysine (BHB-K) (PTM BioLabs; Hangzhou, China).

Plasmids Flag-p53, Flag-p300, CBP-HA and CBP-HA mutants were from Dr. Gu’s lab^4,8^.

siRNA targeting CBP and p300 (1#: 5′-GAGGUCGUUUACAUAAATT-3′; 2#: 5′-UUUAUGUAAACGCGACCUCTT-3′) and a negative control (5′-UUCUCCGAACGUGUCACGUTT-3′) were synthesized by GenePharma (China).

### Cells and cell culture

H1299, 293T and U2OS cells from ATCC were cultured in DMEM (dulbecco’s modified eagle medium), supplemented with 10% FBS (fatal bovine serum). HCT116 cells from ATCC were cultured in MCOY medium supplemented with 10% FBS.

The tetracycline-inducible p53/H1299 cell lines expressing p53 were established by transfecting H1299 cells with a pTRIPZ lentiviral-inducible vector expressing a Flag-p53 fusion protein and selecting the cells with puromycin. After 24 h of transfection, the cells were suspended, diluted and re-seeded to ensure single clone formation. More than 30 clones were selected, and the expression of p53 was evaluated in each clone by western blot with anti-p53 antibodies (DO-1).

Cells were transfected with plasmid DNA or siRNA using Lipofectamine 3000 (Invitrogen) according to the manufacturer’s protocol.

### Protein purification

293T cells were transfected with plasmids containing Flag-p53, Flag-p300 or CBP-HA and cultured for 48 h. The cells were then harvested and lysed in BC500 buffer (20 mM Tris-HCl pH 7.3, 500 mM NaCl, 20% glycerol, 0.5% Triton X-100) with sonication. Anti-Flag M2 beads (for Flag-p53 and Flag-p300) or anti-HA beads (for CBP-HA) were incubated with the cell lysates overnight at 4 °C. The beads were washed with BC100 buffer (20 mM Tris-HCl pH 7.3, 100 mM NaCl, 20% glycerol, 0.1% Triton X-100) three times, and the purified proteins were competitively eluted with Flag peptide (Sigma) or HA peptide (Sigma) in BC100 buffer.

### Detection of p53 kbhb and acetylation inside cells

p53 kbhb was determined in 293T cells transfected with the plasmid expressing Flag-p53 before and after treatment with 10 mM BHB for 24 h. The Flag-p53 fusion protein was purified as previously described but using BC500 buffer supplemented with 10 mM BHB for cells treated with BHB. The purified protein was assayed by western blot using anti-p53 (DO-1) or anti-pan β-hydroxybutyrylation-lysine (BHB-K) antibodies.

Similarly, p53 kbhb was determined in 293T cells cotransfected with both Flag-p53 and CBP-HA plasmids (at a ratio of 1:1) and treated with 10 mM BHB for different times to evaluate the kbhb activity of CBP. The Flag-p53 protein was purified as before and the purified protein was assayed by western blot using anti-p53 (DO-1) or BHB-K or anti-pan-acetylation-lysine (Ac-K) antibodies.

p53 kbhb and acetylation were evaluated in wild-type U2OS cells and HCT116 cells before and after treatment with 10 mM BHB for 24 h. The cells were lysed with RIPA + 0.05% SDS followed by sonication. Lysine β-hydroxybutyrylated and acetylated proteins were purified by immunoprecipitation (IP) with BHB-K and Ac-K antibodies, and p53 was detected by western blots using the anti-p53 antibody (DO-1) with ReliaBLOT® IP/Western Blot Reagent (Bethyl) to inhibit the signal of the Ig heavy chain interference.

### Detection of p53 kbhb in mouse thymus

Six- to eight-week-old C57BL male mice were either fed a standard chow diet or fasted (with free access to water) for 48 h. The mice were euthanized, and their thymus cells were lysed with RIPA + 0.05% SDS followed by sonication. Lysine β-hydroxybutyrylated proteins were purified by IP with BHB-K antibodies, and p53 was detected by western blots with antibodies against full-length p53 diluted in ReliaBLOT® IP/Western Blot Reagent (Bethyl) to inhibit the signal of the Ig heavy chain interference.

### In vitro kbhb assay

The following reactions were prepared in buffer (20 mM HEPES (4-(2-hydroxyethyl)-1-piperazyineethanesulfonic acid), pH 8.0; 1 mM DTT (1,4-dithiothreitol); 1 mM PMSF (phenylmethylsulfonyl fluoride)): 1 µg of p53 and 100 ng of CBP in 0.1 mM β-hydroxybutyrylation-CoA (BHB-CoA); 1 µg of p53, 100 ng of p300 and 0.1 mM BHB-CoA; or 1 µg of mono-nucleosome, 100 ng of CBP and 0.1 mM BHB-CoA. After incubation for 1 h at 30 °C, the reaction products were analyzed by western blot assays with BHB-K antibodies.

### Mass spectrometry assay

Protein complexes were separated by SDS–PAGE and stained with GelCode Blue reagent (Pierce, 24592). The visible band was cut and digested with trypsin and then subjected to liquid chromatography mass spectrometry (LC-MS/MS) analysis.

### Cell growth assay

One hundred and four U2OS cells were spread into six-well plates (three replicates were performed). Cell growth was monitored each day by staining the cells with 2% methylene blue in 50% ethanol for 15 min at room temperature. Stained cells were extracted with 1% SDS and absorbance at 600 nm was measured to quantify cell numbers.

### EdU staining

U2OS cells were treated either with 10 µM Nutlin (Sigma) for 8 h, 10 mM BHB for 16 h, or 10 µM Nutlin for 8 h and 10 mM BHB for 16 h. Or they were treated either with 10 µM doxorubicin for 8 h, or with 10 mM BHB for 16 h, or with 10 µM doxorubicin for 8 h and 10 mM of BHB for 16 h.

The treated cells were then grown in the presence of 50 µM EdU for 2 h, fixed and permeabilized. EdU was used to visualize proliferating cells with a Click-iT® EdU Apollo Fluor® 567 Imaging kit. The cells were then counterstained with DAPI (4',6-diamidio-2-phenylindole) and imaged on a confocal microscope. In all, 20 randomly selected fields were imaged. The number of EdU-positive and DAPI-stained cells were counted and the percentage of cells with EdU-positive labeling was calculated. The experiments were repeated three times. All results are expressed as the mean ± standard deviation. Analysis of variance was performed using Student’s *t* test to determine the statistical significance of differences among the groups.

### Apoptosis

HCT116 cells were treated with either 10 µM Nutlin for 8 h, 10 mM BHB for 16 h, or 10 µM Nutlin for 8 h and 10 mM BHB for 16 h, and apoptotic cells were identified using a FITC-Annexin V Staining kit following the manufacturer’s instructions.

Six- to eight-week-old C57BL male mice (five mice per group) were either fed a standard chow diet or fasted with free access to water for 48 h. The mice were treated with 12.5 Gy of γ-irradiation and euthanized 4 h later. Fed mice were used as controls. Thymus cells were obtained from all four groups. Apoptotic cells stained with FITC-conjugated Annexin V and necrotic cells stained with propidium iodide were analyzed by flow cytometry.

### Reverse transcription quantitative PCR (RT-qPCR)

Total RNA was extracted from mouse thymus tissue using TRIzol (Invitrogen) according to the manufacturer’s protocol. Two micrograms of total RNA was reverse-transcribed into cDNA using SuperScript III First-Strand Synthesis SuperMix (Invitrogen). The relative expression level of each target gene was measured by qPCR and normalized to the level of actin. We focused on genes targeted by p53^4,8^. The primers used are listed in Supplementary Table S1.

### *p21* and *PUMA* expression in mice

Six- to eight-week-old C57BL male mice (each group consisted of five mice) were either fed a standard chow diet or fasted with free access to water for 48 h. The mice were treated with 12.5 Gy of γ-irradiation and euthanized 4 h later. Untreated mice were used as controls. Thymus cells were lysed, and p21 and PUMA expression were analyzed by immunoblotting.

### Measurement of BHB in mouse serum

Six- to eight-week-old C57BL male mice (each group consisted of five mice) were either fed a standard chow diet (five mice) or fasted with free access to water (five mice) for 48 h. Blood samples were taken from a tail vein for determination of blood BHB concentrations using a ketone meter (China, Alibaba).

### Mice

Six- to eight-week-old C57BL male mice were licensed by Beijing Municipal Committee of Science and Technology. All mice were housed in a temperature-controlled room (22 ± 2 °C) with a light/dark cycle of 12 h/12 h. All animal experiments were performed according to Wenhui Zhao’s animal protocols (No. LA2018013) approved by the Animal Ethics Committee of the Peking University Health Science Center, China.

### Statistical analysis

All the experiments were performed at least in triplicate, and all results are expressed as the mean ± standard deviation. All mouse data represent the average of results obtained in five 6- to 8-week-old male mice. Analysis of variance was performed using the Student’s *t* test to determine the statistical significance of differences among the groups.

## Results

### p53 kbhb occurs in cells in the presence of BHB and in the thymus of fasted mice

**Fig. 1:**
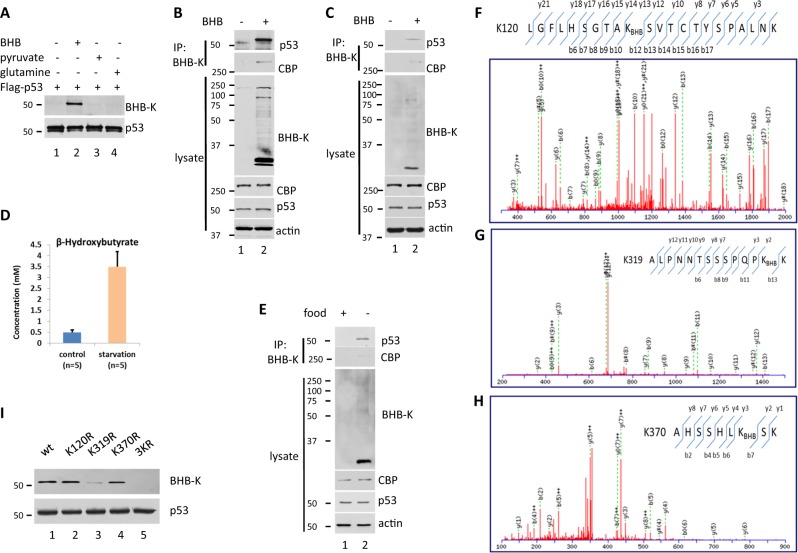
Identification and characterization of β-hydroxybutyrylation (kbhb) modification of p53. **a** kbhb modification of overexpressed p53 in 293T cells. Flag-p53 was purified from 293T cells treated with 10 mM β-hydroxybutyrate (BHB), pyruvate or glutamine for 24 h. The purified protein was detected by western blot using anti-pan β-hydroxybutyrylation-lysine antibodies (BHB-K) or anti-p53 (DO-1). **b** Kbhb modification of endogenous p53 in U2OS cells. Western blot with p53 (DO-1), BHB-K or actin antibodies of cell lysates of U2OS cells treated with 10 mM BHB (bottom half, labeled as “lysate”) or cell lysates immunoprecipitated (IP) with BHB-K antibodies (top, IP: BHB-K) with ReliaBLOT® IP/Western Blot Reagent (Bethyl) to inhibit the signal of the Ig heavy chain interference. **c** Kbhb modification of endogenous p53 in HCT116 cells. Cell lysates of HCT116 cells treated with or without 10 mM BHB for 24 h were IP with BHB-K antibodies. Western blot analysis of p53 by DO-1 antibodies as in (**b**). **d** The BHB in mouse serum were measured. Mice were fasted for 48 h or fed with a normal diet. Blood samples were taken from a tail vein for determination of blood BHB concentrations using a ketone meter. **e** Kbhb modification of endogenous p53 in mice thymus tissue. Thymus cell lysates of mice fasted for 48 h or fed with a normal diet were IP with BHB-K antibodies. Western blot analysis of p53 with anti-full-length p53 antibodies as in (**b**). **f−h** Mass spectrometry analysis identified p53-derived peptides containing β-hydroxybutyrylated Lys-120, Lys-319, and Lys-370. p53 purified from 293T cells transfected with plasmid DNA expressing Flag-p53 and treated with 10 mM BHB for 24 h was analyzed by HPLC-MS/MS. Annotated MS/MS spectra of peptides LGFLHSGTAKSVTCTYSPALMK (derived from amino acids 111−132, **f**), ALNTSSSPQPKK (derived from amino acids 307−320, **g**) and AHSSSHLKSK (derived from amino acids 364−372, **h**) was showing as b- and y-ion coverage. **i** Effect of lysine-to-arginine mutation on p53 kbhb. Western blot analysis using BHB-K of wild-type and mutant Flag-p53 expressed in 293T cells treated with 10 mM BHB for 24 h

To explore whether kbhb occurs in p53, first, we purified and enriched this protein from 293T cells expressing Flag-tagged p53 and treated with BHB, pyruvate or glutamine. Immunoblot analysis was performed with anti-p53 and anti-pan β-hydroxybutyrylation-lysine antibodies (BHB-K), and the results showed that the purified p53 was recognized by both antibodies in cell lysates from cells treated with BHB but not in those from cells treated with pyruvate or glutamine, thus suggesting that p53 is modified by Kbhb in the presence of BHB, but not in the presence of pyruvate or glutamine (Fig. 1a).

Then, we assessed whether kbhb occurs in endogenous p53 in U2OS cells whose p53 wild-type and signal pathway are complete. To this end we purified and enriched the Kbhb proteins from BHB-treated or -untreated U2OS cells by IP using the BHB-K antibody and the p53 protein was detected by western blot. As shown in Fig. 1b, the levels of p53 were notably higher in Kbhb-enriched lysates from BHB-treated cells than in those of untreated cells, while the levels of p53 remained unchanged in total cell lysates regardless of the treatment (Fig. 1b). This result was further confirmed in HCT116 cells, which are another kind of cells with wild-type p53 and a complete p53 signal pathway (Fig. 1c).

Finally, we assessed whether the cellular p53 Kbhb levels increase in response to high levels of BHB under physiological conditions. To this end, we determined p53 Kbhb in the thymus tissue of C57BL mice that were fed a normal chow diet or subjected to 48 h of fasting (supplied with water only). Serum BHB concentrations were higher sevenfold in the fasted mice than in the controls fed a normal diet (Fig. 1d), same as the literature described^13^. In addition, the levels of p53 were substantially higher in thymus cell lysates from fasted mice enriched by immunoprecipitation with the BHB-K antibody than in those from normal-fed mice (Fig. 1e). By contrast, the levels of p53 in total cell lysates were similar regardless of the diet (Fig. 1e).

Together, our results indicate that p53 undergoes kbhb.

### p53 kbhb occurs at lysines 120, 319, and 370

To confirm the presence of Kbhb in p53 and determine the site of kbhb, we purified Flag-p53 from transfected 293T cells that were treated with BHB, and analyzed the purified protein by HPLC-MS/MS. The mass spectrometry data showed that there was a mass shift at the lysine 120 residue of the p53 peptide, LGFLHSGTAKBHBSVTCTYSPACAK, which indicated that kbhb had occurred at lysine 120 (Fig. 1f). The mass spectrometric analysis also revealed that the lysine residues 319 and 370 were also modified (Fig. 1g, h). To validate these findings, we transfected 293T cells with plasmids expressing Flag-tagged p53 proteins with one or all three lysines mutated to arginine (120R/p53, K319R/p53, K370R/p53, and K120,K319,K370R/p53 (3KR p53), respectively) and we incubated the cells in the presence of BHB. Western blot analysis of the purified p53 using BHB-K antibodies showed that the signal was lower in K319R mutant and completely disappeared in the triple mutant (Fig. 1i), suggesting that the kbhb modification sites were at lysines 120, 319, and 370. Together, our results indicate that p53 undergoes kbhb at these three lysine residues.

### p53 kbhb is catalyzed by CBP

In addition to lysine acetylation, it was recently shown that various short-chain lysines undergo acylation in core histones, including lysine propionylation, butyrlation, 2-hydroxyisobutyrylation, 3-hydroxybutyrylation, crotonylation, malonylation, succinylation, and glutarylation^13,25–29^. It is well-known that CBP/p300 is a histone acetyltransferase that acetylates p53 at K98 and K101^30^, K164^31^ and also six lysines of the C-terminal region^4,32–35^. Moreover, p300 was recently identified as a histone butyryltransferase that catalyzes histone butyrylation^33,34^.

To test the possibility that CBP is a β-hydroxybutyryl-transferase that catalyzes the kbhb of p53, we first investigated whether kbhb occurs in CBP. Interestingly, when purifying and exploring the Kbhb proteins from U2OS and HCT116 p53^+/+^ cell lines by IP using the BHB-K antibody, we can clearly detect the presence of CBP protein in the BHB treatment group without the protein expression level of CBP (Fig. 1b, c). Furthermore, the same results were found that more CBP was detected in the group of the fasted mice whose thymus lysates were enriched with BHB-K antibody in contrast to those of normal-fed mice (Fig. 1e).

**Fig. 2:**
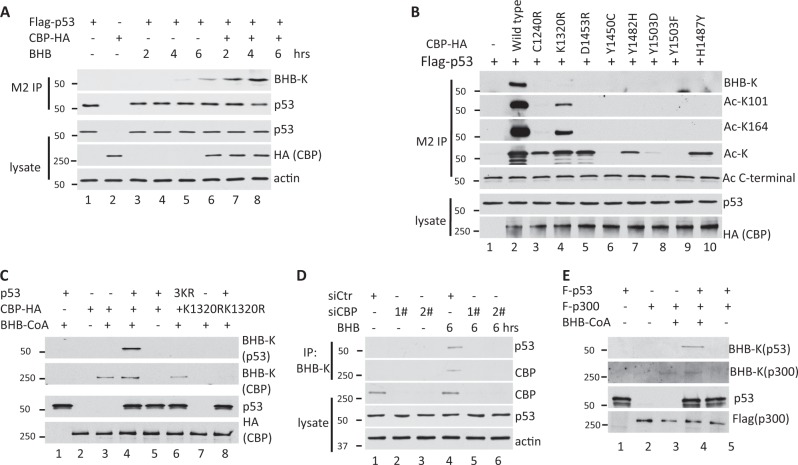
CBP β-hydroxybutyrylates p53 inside cells and in vitro. **a** p53 kbhb in the presence or absence of BHB in cells. Western blot analysis of p53. IP was performed using BHB-K antibodies in cells transfected with p53 or both p53 and CBP and then treated with 10 mM BHB. **b** CBP mutants lacked the ability to exert kbhb activity on p53. Western blot analysis of p53 performed using antibodies against BHB-K, Ac-K and specific acetylated sites on p53 in IP obtained from cells transfected with p53, p53 plus wild-type CBP, or p53 plus mutant CBP and then treated with 10 mM BHB, 1 μM TSA and 5 mM nicotinamide for 24 h. **c** CBP β-hydroxybutyrylates p53 in vitro. Purified p53 was incubated with CBP or CBP mutants and β-hydroxybutyrate-CoA (BHB-CoA). Western blot analysis of the reaction products performed with BHB-K antibodies (top two panels), with anti-p53 (p53 labeled panel) or with anti-HA antibody (bottom panel to detect CBP). **d** Silencing CBP reduced the β-hydroxybutyration level of p53 in U2OS. Cells was transfected twice with 120 pmol of siRNA oligos specially targeting CBP and p300 or negative control, followed by treating with 10 mM BHB after 48 h. **e** p300 β-hydroxybutyrylates p53 in vitro. Purified p53 was incubated with p300 and BHB-CoA. Western blot analysis of the reaction products performed with BHB-K antibodies (top two panels), with anti-p53 (p53 labeled panel) or with anti-Flag antibody (bottom panel to detect p300)

To further confirm whether CBP is a β-hydroxybutyryl-transferase of p53, we purified Flag-p53 from 293T cells expressing CBP and treated with BHB for 2−6 h. Western blot analysis with BHB-K antibody showed that the levels of p53 Kbhb increased over time and in the presence of CBP (Fig. 2a). These results suggest that CBP might be the β-hydroxybutyryl-transferase that catalyzes p53 kbhb.

**Fig. 3:**
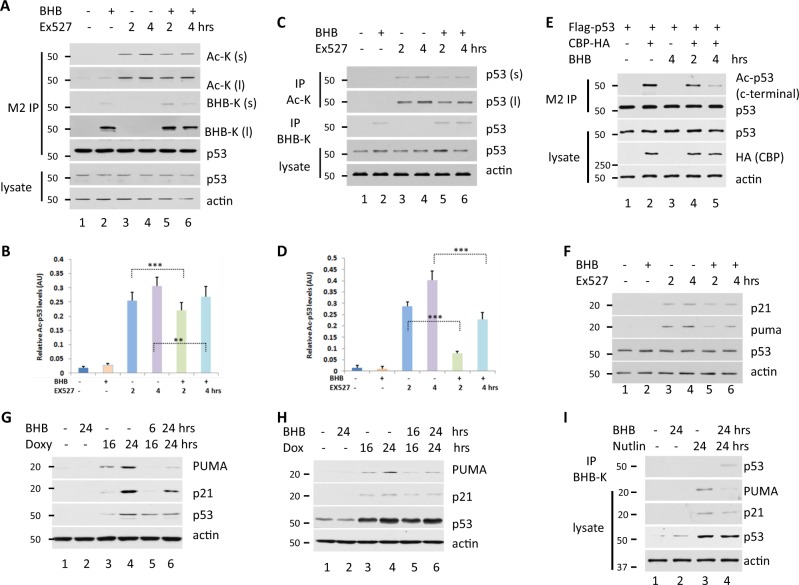
kbhb reduces the acetylation of p53. **a** H1299 cells were transfected with Flag-p53 and then treated with 10 mM BHB for 4 h, with 10 µM Ex527 (an inhibitor of SIRT1), for 2 or 4 h, or with both BHB and Ex527. p53 was purified and enriched by IP. Western blot analysis using BHB-K and Ac-K antibodies of purified p53 (M2 IP) or whole cell lysates (lysate) with different exposure time (s: shorter exposure time; l: longer exposure time). **b** Quantification of acetylation p53 protein levels to normalize to p53 protein levels by western blot represented in (**a**). Data shown are averages of three times + SEM. The *p* value was determined by paired Student’s *t* test (**p < 0.005, ***p < 0.001). **c** HCT116 cells were treated with 10 mM BHB for 4 h, 10 µM Ex527 for 2 or 4 h, or with both BHB and Ex527. Cell lysates were IP with BHB-K or Ac-K antibodies. Western blot using anti-p53 DO-1 antibodies of Ac-K and BHB-K IP, or of the whole cell lysate with different exposure time (s: shorter exposure time; l: longer exposure time). **d** Quantification of acetylation p53 protein levels to normalize to p53 protein levels by western blot represented in (**c**). Data shown are averages of three times + SEM. The *p* value was determined by paired Student’s t test (***p < 0.001). **e** Western blot analysis to determine the level of acetylated p53 using Ac-p53 antibodies, anti-p53 in p53-purified IP obtained from cells transfected with p53 or both p53 and CBP and then treated with 10 mM BHB for the indicated time. IP were performed with M2 beads. Western blot of whole cell lysates using anti-p53, anti-HA (to detect CBP) and anti-actin is also shown (bottom). **f** HCT116 cells were treated with 10 mM BHB for 4 h, 10 µM Ex527 for 2 or 4 h, or both BHB and Ex527. The expression levels of p53, actin (as a loading control) and the p53 downstream genes p21 and PUMA were analyzed by western blot. **g** The levels of the p53 downstream genes p21 and PUMA were decreased in the p53/H1299-inducible cell lines regardless of whether they were untreated, treated with BHB, treated with doxycycline to induce p53 expression, or treated with BHB and doxycycline together. **h** p21 and PUMA expression were decreased in HCT116 cells treated with BHB, Doxorubicin to damage DNA to activate p53, or both BHB and Doxorubicin. **i** BHB treatment decreased the p21 and PUMA expression induced by nutlin in U2OS cells

To further confirm whether  the role of CBP on p53 kbhb, we assessed p53 kbhb and acetylation in Flag-p53-expressing 293T cells transfected with plasmids expressing wild-type CBP or CBP with various mutations that have been associated with a loss of CBP’s p53 acetyltransferase activity in lymphomas^35^. We hypothesized that mutations in CBP might modulate its ability to act as a β-hydroxybutyryl-transferase to catalyze the kbhb of p53. We transfected 293T cells with plasmids expressing Flag-tagged p53, wild-type CBP, or CBP with various mutations that are observed in lymphomas. Flag-p53 purified from the 293T cells treated with BHB and two deacetylation inhibitors was analyzed by western blot using antibodies that recognize the acetylation of p53 K101, K164, or the C-terminal lysines, pan-acetylation of lysine (Ac-K), as well as with BHB-K. The results showed that all of the cells transfected with CBP mutation-containing plasmids lost their ability to β-hydroxybutyrylate p53 (panel BHB-K in Fig. 3b), while the K1320R mutant CBP maintained its ability to acetylate K101, K164 and the C-terminal region of p53. K1240R CBP, D1453R CBP, Y1482H CBP and H1487Y CBP still acetylated p53 at its C-terminal region, and only Y1450C CBP, Y1503D CBP and Y1503F CBP lost their ability to acetylate p53 (Fig. 2b). These results further support a role for CBP in p53 kbhb.

Next, we tested whether CBP could β-hydroxybutyrylate p53 in vitro using purified p53 and CBP proteins and BHB-CoA as a co-factor (Fig. 2c). We found that a kbhb signal was only detected for p53 when wild-type CBP and p53 were incubated in the presence of BHB-CoA, but not when a mutant CBP was used (lanes 4 vs. 8) or when the 3KR p53 mutant was used (lanes 4 vs. 6). We found that a kbhb signal was detected for CBP when wild-type (lanes 3, 4, and 6), but not mutant (lanes 7 and 8), CBP was incubated with BHB-CoA, no matter with or without p53 or 3KR p53, which shows that CBP catalyzes itself with β-hydroxybutyryl-transferase activity, similar to CBP acetylation (Fig. 2c).

To better understand the potential role of CBP on the β-hydroxybutyrylation of p53, two different siRNA oligos were used to knock down the CBP in U2OS cell lines, and we found that CBP protein expression level was significantly reduced compared to the negative control. Consistent with the expected results, the CBP knockdown expression caused a lower level of the β-hydroxybutyrylation of p53 (Fig. 2d). Those results further supported that CBP can β-hydroxybutyrylate p53.

Because CBP and p300 are very similar protein sequences and their structure even functions, and both are acetyltransferases, we tested whether p300 could β-hydroxybutyrylate p53. We set up an in vitro assay using purified p53 and p300 proteins and BHB-CoA as a cofactor (Fig. 2e). The results demonstrated that p300 can act as a β-hydroxybutyryl-transferase for p53 and p300 themselves, and works similar to CBP, which are the same as their acetyltransferase activity (Fig. 2d).

Together, our results indicate that p53 is a bona fide kbhb substrate of CBP/p300 both in vivo and in vitro.

### Kbhb reduces p53 acetylation and activation

We identified p53 Kbhb, in addition to p53 lysine acetylation, both of which are short-chain lysine acylations. Because the same enzyme (CBP) catalyzes both types of p53 modification, and because kbhb affects the acetylation of histones in previously described cases^13^, we wanted to study whether p53 Kbhb might affect p53 acetylation under different conditions. To investigate this, Flag-p53-expressing 293T cells were treated with BHB and/or an inhibitor of the deacetylase SIRT1 (Ex527) and acetylation and kbhb were assessed in purified p53 by immunoblotting with Ac-K and BHB-K antibodies, respectively (Fig. 3a, b). The results showed that the levels of p53 acetylation were lower in the presence of BHB when p53 Kbhb was detected (Fig. 3a, b). Similar results were obtained in HCT116 cells (Fig. 3c, d). These results were more evident when CBP was overexpressed in the presence and absence of BHB in 293T cells. The levels of p53 acetylation in CBP-overexpressing cells were remarkably lower in BHB-treated cells than in untreated cells (Fig. 3e). These results suggested that BHB treatment induced the β-hydroxybutyrylation of p53 and preferentially interfered with the acetylation of p53 c-terminal in cells.

Since posttranslational modification of p53 is known to affect its activity^2–8^, we next investigated whether p53 kbhb also influences p53 activity. To this end, HCT116 cells were treated with Ex527 and/or BHB, and the expression of the classic p53 target gene upregulated the modulator of apoptosis (PUMA, also known as BCL2 binding component 3, BBC3), and the cyclin-dependent kinase inhibitor 1A (CDKN1A, also known as p21) was analyzed at the protein level by western blot (Fig. 3f). As expected, p53 acetylation levels increased with Ex527 treatment and p21 and PUMA expression was upregulated (Fig. 3f). However, when cells were simultaneously treated with BHB and Ex527, p21 and PUMA protein expression levels were lower than in Ex527-only treated cells (Fig. 3f). These results suggest that under conditions that favor p53 kbhb over acetylation, p53 gene regulatory activity is attenuated.

Next, we investigated the effect of kbhb on p53 activity by detecting p21 and PUMA expression in p53-inducible expressing H1299 cells treated with the doxycycline to induce p53 expression and/or BHB. As expected, doxycycline treatment induced p53 expression and that of the p53-regulated genes p21 and PUMA at the protein level. However, the protein levels of p21 and PUMA were much lower when the cells were coincubated with BHB (Fig. 3g).

Last, we investigated the effect of kbhb on p53 activity under genotoxic stress by detecting p21 and PUMA expression in HCT116 cells treated with the DNA-damaging agent doxorubicin and/or BHB (Fig. 3h). Similar results were obtained as in the induction of p53-expressing H1299 cells; doxorubicin treatment activated p53 and upregulated genes p21 and PUMA at the protein level. However, the protein levels of p21 and PUMA were much lower when the cells were coincubated with BHB (Fig. 3h).

Similar results of Kbhb on endogenous p53 activity, nutlin, an MDM2 inhibitor, was used to increase the level of endogenous p53 protein in U2OS. The results showed that nutlin can significantly increase the level of endogenous p53 protein and increase the protein expression levels of p21 and PUMA (line 3), but the protein expression levels of p21 and PUMA are significantly downregulated in combination with BHB treatment (line 4, Fig. 3i). These results show that the β-hydroxybutyrylation of p53 can alter the acetylation of p53 and affect the transcriptional activity of p53 on its target gene.

### p53 cell growth arrest and apoptosis-inducing functions are attenuated in the BHB-treated cells

**Fig. 4:**
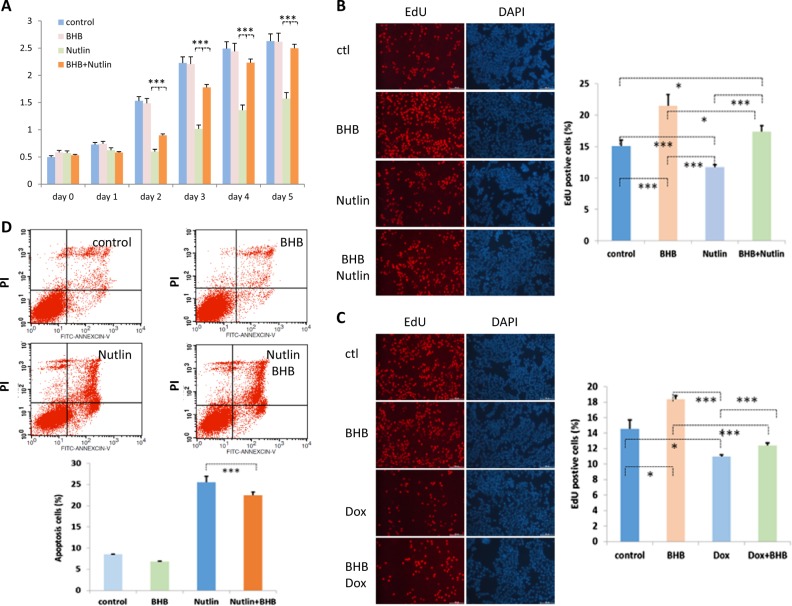
p53 activity is attenuated in β-hydroxybutyrate-treated cells. **a** Cell growth curve analysis of U2OS cells. A total of 3×10^4^ U2OS cells were seeded into six-well plates on day 1. The cells were left untreated, or treated with 10 mM BHB, 10 µM Nutlin, or both. Cell growth was monitored each day by staining the cells with 2% methylene blue in 50% ethanol for 15 min at room temperature. Stained cells were extracted with 1% SDS and the OD was measured at 640 nm. Data shown are averages + SEM. The *p* value was determined by paired Student’s *t* test (***p < 0.001). **b** U2OS cells were treated with 10 µM Nutlin for 8 h, 10 mM BHB for 16 h, or both. The treated cells were then grown in the presence of 50 µM EdU for 2 h, fixed and permeabilized. The proliferating cells were visualized using a Click-iT® EdU Apollo Fluor® 567 Imaging kit. The EdU-positive cells were counted under a microscope in 20 randomly chosen fields, and the percent was calculated. The experiments were carried out three times, and the averages of the data are presented. Cells were counterstained with DAPI. Data shown are averages + SEM. The *p* value was determined by paired Student’s *t* test (*p < 0.05, ***p < 0.001). **c** U2OS cells were treated with 10 mM BHB for 16 h, 10 µM doxorubicin for 8 h or both, and cell proliferation was measured as described in (**b**). Data shown are averages + SEM. The *p* value was determined by paired Student’s *t* test (*p < 0.05, ***p < 0.001). **d** FACS analysis of apoptosis in HCT116 cells treated with 10 µM Nutlin for 8 h, 10 mM BHB for 16 h or with both BHB and Nutlin. Apoptotic cells were stained with FITC-conjugated Annexin V and counterstained with propidium iodide. The cells were analyzed by FACS. Data shown are averages + SEM. The *p* value was determined by paired Student’s *t* test (***p < 0.001)

Since p53 is involved in the regulation of cell growth and cell apoptosis and this is particularly important in tumor development, we decided to study how kbhb affected the cell growth arrest function and cell apoptosis function of p53. To this end we monitored the cell growth of U2OS cells treated with BHB and/or Nutlin, a compound that prevents MDM2 from degrading p53, thus resulting in p53 stabilization and activation. As expected, Nutlin-treated cells grew more slowly than control cells or cells treated with BHB alone (Fig. 4a). By contrast, cells treated with both Nutlin and BHB grew at a similar rate to control cells and cells treated with BHB only (Fig. 4a). Similar results were obtained in U2OS cells treated with Nutlin and/or BHB, after measuring cell growth using EdU staining (Fig. 4b).

We also assayed cell proliferation by EdU staining in U2OS cells treated with BHB and/or the DNA-damaging agent doxorubicin that damage DNA to induce p53 activation and, therefore, cell growth arrest. The results showed that the percentage of EdU-positive cells was lower when cells were treated with doxorubicin, relative to untreated controls, as expected. However, when cells were treated with both doxorubicin and BHB, the percentage of EdU-positive cells increased, relative to cells treated with doxorubicin alone (Fig. 4c). Together, these results indicate that under p53 kbhb-inducing conditions, the cell growth arrest function of p53 is attenuated.

In addition, we investigated the effect of kbhb on the apoptosis-inducing function of p53 in HCT116 cells treated with Nutlin (Fig. 4d). The results showed a small but significant reduction in the apoptosis of HCT116 cells in the presence of BHB compared to that of cells treated with Nutlin alone (Fig. 4d), supporting a reduction of p53 activity under kbhb-inducing conditions.

### Induction of p53-regulated genes is reduced in tissues of fasted mice under genotoxic conditions

**Fig. 5:**
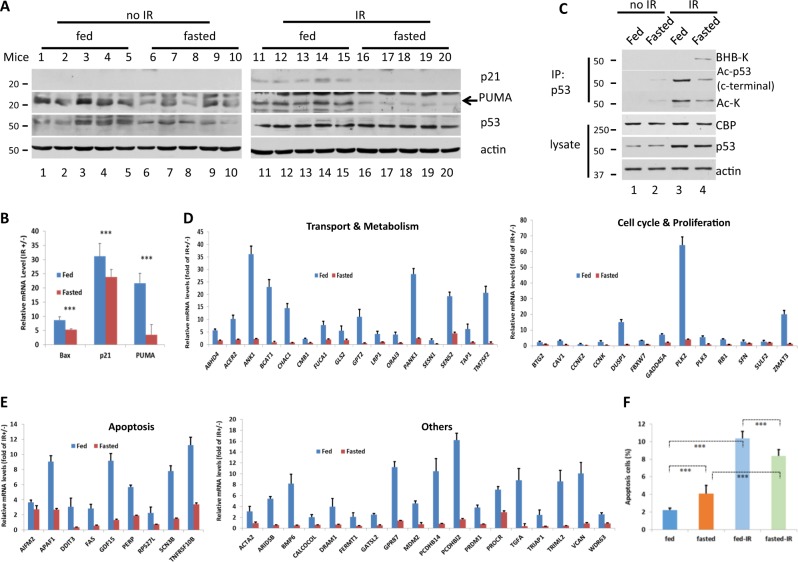
β-hydroxybutyrylated p53 inhibits downstream gene expression. Six- to eight-week-old C57BL male mice (*n* = 5 in each group) were fed a standard chow diet or fasted (with free access to water) for 48 h and exposed to 12.5 Gy of γ-irradiation or left untreated. Then, 4 h later, the mice were euthanized, and the thymus was collected. **a** Thymus cells were lysed, and p21 and PUMA protein levels were analyzed by immunoblot. **b** Thymus RNA was extracted and mRNA expression was measured by RT-qPCR. The starvation mice had lower levels of Bax, p21 and PUMA RNA, but these levels were upregulated by γ-irradiation to damage DNA, which induced p53 activation. **c** kbhb affected the acetylation level of p53 in vivo. Thymus lysates were IP with p53 (full-length) antibody and western blot using Ac-p53 (c-terminal), Ac-K or BHB-K antibody. **d**, **e** The starvation mice showed less upregulation of p53 downstream genes in response to γ-irradiation to damage DNA, which induces p53 activation. All levels of significance are at least *p* < 0.01. **f** Thymus cell apoptosis was assayed using a FITC-Annexin V kit. The rate of apoptosis was lower in the thymus tissues of fasted mice than in those of fed mice in 12.5 Gy γ-irradiation-treated animals even though in nonirradiated animals, the rate of apoptosis was higher in the thymus tissues of fasted mice than in fed mice. All results are expressed as the mean ± standard deviation. All data represent the average of five mice and triplicate experiments performed in each mouse. Analysis of variance was performed with the Student’s *t* test to determine the level of statistical significance of differences among groups. A value of *p* < 0.05 was considered statistically significant (*p < 0.05, **p < 0.01, ***p < 0.001)

To explore the effect of p53 Kbhb in vivo, we analyzed thymus tissues obtained from fasted C57BL mice under genotoxic conditions. Mice were fed with a normal chow diet or subjected to 48 h of fasting (supplied with water only), and were then γ-irradiated to damage their DNA, therefore inducing p53 activation. Mice blood BHB was increased about sevenfold of fasted mice than fed, to induce p53 Kbhb (Fig. 1d, e). p53 activity was monitored by following the expression of its downstream target genes PUMA and p21 in the thymus at the protein level. In nonirradiated mice, PUMA protein levels were lower in fasted mice than in fed mice, while the levels of p21 were below the limit of detection (Fig. 5a). By contrast, p21 could be detected in the γ-irradiated group where the levels of both p21 and PUMA were higher than in the nonirradiated group, regardless of whether the mice were fed or fasted (Fig. 5a). In addition, within the mice exposed to γ-irradiation, the levels of p21 and PUMA were lower in the fasted than in the fed mice (Fig. 5a).

Expression of *p21* and *PUMA* was also assessed at the mRNA level in the thymus tissues of the fasted and fed mice exposed or unexposed to γ-irradiation. The expression of these genes was significantly increased by γ-irradiation (*p* ≤ 0.001), as expected. However, the extent of this increase was much larger in the fed than in the fasted mice (Fig. 5b). Similar results were obtained for the p53 target gene Bax (Fig. 5b).

To confirm that the expression pattern changes of *p21* and *PUMA* between the fasted and fed mice were caused by the β-hydroxybutyrylation of p53, both β-hydroxybutyrylation and acetylation levels of p53 in the mice were examined. We found that IR can cause a significant increase in p53 protein levels in the thymus, no matter normal fed or fasted treatment; while the expression level of CBP was not significantly altered in all the treatments (Fig. 5c). Furthermore, a fasted treatment in IR mice resulted in a significant reduction in the level of acetylation of p53 compared to the normal diet group (Fig. 5c, lines 4 vs. 3), and in the no-irradiated mice, the same fasted treatment resulted in a slight increase in the level of acetylation of p53 (Fig. 5c, lines 2 vs. 1). To conclude, we found that starvation results in increasing BHB levels in plasmas (Fig. 1d), leading to an increase in the β-hydroxybutyrylation of p53 in the thymus with a decrease in the acetylation of p53, which causes a change in the expression pattern of *p21* and *PUMA*, which is consistent with the in vitro data (Fig. 3).

To further validate the effect of p53 Kbhb, we next extended to investigate the expression of additional p53 target genes identified in the literature^21,22^. The results showed that in fed mice, as expected, all the expression of all the assessed genes considerably increased by γ-irradiation (Fig. 5d, e). However, the results showed that in all the cases the induction by γ-irradiation was lower in fasted mice compared to fed mice with statistically significant differences (Fig. 5d, e).

Finally, we investigated the effects of γ-irradiation on the apoptosis of cells from the thymus tissues of fed and fasted mice (Fig. 5f). Nonirradiated mice, both fed and fasted, had significantly fewer apoptotic cells in their thymus tissues than did the fed and fasted mice exposed to 12.5 Gy of γ-irradiation (Fig. 5f). In addition, in nonirradiated mice, there were fewer apoptotic cells in the thymus of the fed mice relative to the fasted mice (Fig. 5f). By contrast, in γ-irradiated mice the percentage of apoptotic cells was lower in fasted than in fed mice (Fig. 5f).

Overall, these results indicate that under kbhb conditions there is a reduced genotoxic-induced p53 activation resulting in reduced expression of p53-regulated genes and reduced cell apoptosis.

## Discussion

p53 is an essential tumor suppressor. The TP53 gene (in mice it is Trp53 gene) has long been recognized as a vitally important tumor suppressor gene because it is mutated and inactivated in more than 80% of human cancer cases^1^. The p53 protein is one of the most widely studied transcription factors. It has been found that p53 activity is finely tuned by the posttranslational modifications such as acetylation, methylation, phosphorylation, ubiquitination, sumoylation, and neddylation^2–4^.

The major ketone bodies are BHB and acetoacetate, which can be converted into each other and trace amounts of acetone. Ketone bodies are predominantly formed in the liver by acetyl coenzyme A, which degrades fatty acids via β-oxidation. In addition to serving as an energy source, ketone bodies are also recognized as factors that fulfill signaling roles in cellular homeostasis^12–17^ (reviewed in refs. ^12–14^). BHB plays signaling roles by a mechanism that BHB induces kbhb, which is a novel histone posttranslational modification^13^.

Kbhb has been described only in histone proteins. Our prior evidence from mass spectrometry analysis data suggested that p53 may be β-hydroxybutyrylated. Therefore, here we studied p53 kbhb. p53 kbhb is an entirely novel discovery.

We have found for the first time that p53 can be modified by kbhb in the presence of BHB and that this modification is catalyzed by the acetyltransferase CBP. In particular, our results demonstrate that p53 Kbhb occurs in cells treated with BHB and in the thymus tissues of fasted mice, in which the serum concentrations of BHB increased. In addition, we found that in the presence of BHB the levels of p53 acetylation, which correlate with p53 activity^13,27–29^, were reduced in favor of p53 kbhb. Consistent with these results, our findings show that the upregulation of p53 target genes by genotoxic stress was lower in fasted mice than in fed mice. In addition, apoptosis and cell growth arrest mediated by p53 activated by DNA damage or protected from degradation by Nutlin, were decreased by BHB treatment.

Ketone bodies, such as BHB, are reported to play a role in cancer biology^21–24^; however, their specific role remains to be elucidated. It has been reported that ketone bodies reduced pancreatic cancer growth in mouse xenograft models^21^, and decreased the proliferation and viability of the highly metastatic VM-M3 cells, and prolonged the survival of VM-M3 xenograft mice^22^. On the contrary, the ketone body acetoacetate has been found to drive tumor cell proliferation and growth in melanoma through a cellular signaling pathway involving MEK-ERK signaling^23^. The use of ketogenic diets and calorie restriction also have therapeutic effects in human and mouse brain tumors^24^. But it has been recently reported that ketogenic diets only changed the flora of bacterium in the digest system, not ketone bodies to contribute the therapeutic effects^36^. Our findings show that p53 Kbhb is induced by BHB and that kbhb attenuates p53 activity. Since p53 is an important tumor suppressor protein, attenuation of its activity in the presence of BHB might partially explain the role of ketone bodies in cancer. The inconsistency of the previous report may be because that p53 mutation has not been taken into consideration.

ur findings show that BHB induces p53 kbhb. Fatty acid metabolism via β-oxidation produces acetyl coenzyme A to synthesize ketone bodies in the liver, which spread to the whole body. Therefore, we can view from a different angle the relationship between fatty acids and cancer. We paved a new way to explore the function of fatty acids metabolism in cancer, which need to be studied deeply in the future.

We found that CBP β-hydroxybutyrylate histones and p53, in addition to acetylating histones and p53^7–9^. From our point of view, this may be caused by a sharp rise in the level of BHB-CoA in cells treated with BHB. This concentration advantage induces CBP to catalyze the β-hydroxybutyrylation of p53, which in turn affects CBP to catalyze the acetylation of p53. Thus, CBP appears to be a flexible enzyme that might play important roles in acylation, which need to be further investigated in future studies.

These data suggest that CBP β-hydroxybutyryl-transferase activity is more strictly limited than is acetyltransferase activity on p53. However, this might be because our BHB-K antibody is not as sensitive at detecting lower levels of p53 Kbhb as are other site-specific acetylation antibodies, such as those for K101, K164 and the C-terminal region of p53. This issue requires further investigation with site-specific BHB-K antibodies.

## Supplementary information

Real time PCR primers
